# Myocarditis, Myositis, and Myasthenia Gravis Overlap Syndrome Associated with Immune Checkpoint Inhibitors: A Systematic Review

**DOI:** 10.3390/diagnostics14161794

**Published:** 2024-08-16

**Authors:** Demis N. Lipe, Aiham Qdaisat, Pavitra P. Krishnamani, Trung D. Nguyen, Patrick Chaftari, Nour El Messiri, Aswin Srinivasan, Elkin Galvis-Carvajal, Cielito C. Reyes-Gibby, Monica K. Wattana

**Affiliations:** 1 ProPharma Group, Raleigh, NC 27601, USA; 2 Department of Emergency Medicine, The University of Texas MD Anderson Cancer Center, Houston, TX 77030, USA; aqdaisat@mdanderson.org (A.Q.);; 3 Department of Cardiology, HCA Houston Kingwood, College of Medicine, University of Houston, Kingwood, TX 77339, USA; 4 Department of Emergency Medicine, Baylor College of Medicine, Houston, TX 77030, USA

**Keywords:** immunotherapy, immune-related adverse events, myasthenia gravis, myocarditis, myositis, immune checkpoint inhibitors, cardiovascular

## Abstract

Immune checkpoint inhibitors (ICIs) have significantly transformed cancer treatment, but their use is linked to immune-related adverse events (irAEs), including the rare ICI-associated myocarditis, myositis, and myasthenia gravis (MMM) overlap syndrome. This systematic review aims to highlight MMM’s clinical implications in emergency departments. PubMed and Embase were searched using a specific search strategy. Reports were eligible for inclusion if all three conditions were present and associated with the use of an ICI. Data were extracted by independent reviewers using the Rayyan web application for systematic reviews. Descriptive statistics and qualitative synthesis were used to summarize demographic, clinical, and treatment data for the reported cases. Among 50 cases, predominantly associated with melanoma, lung cancer, and renal cancer, the in-hospital mortality rate was 38.0%. The most commonly presenting symptoms were ptosis (58%), dyspnea (48%), diplopia (42%), or myalgia (36%). The median time from ICI initiation to MMM presentation was 21 days (interquartile range: 15–28 days). Corticosteroids were the primary treatment for the irAEs. MMM, a rare but potentially fatal complication of ICI therapy, requires prompt recognition in emergency settings. Corticosteroids should be initiated if suspected, without waiting for confirmation. Multidisciplinary collaboration is vital for diagnosis and treatment planning. Research on MMM’s link to specific cancers and ICIs is imperative for better risk assessment and interventions.

## 1. Introduction

Over the past twenty years, there has been a steady and significant increase in the use of immunotherapy as a treatment modality for a wide range of malignancies. Notably, immune checkpoint inhibitors (ICIs) including antiprogrammed cell death-1 (PD-1), antiprogrammed cell death ligand-1 (PD-L1), anticytotoxic T-lymphocyte antigen-4 (CTLA-4) and lymphocyte activation gene-3 (LAG-3) such as nivolumab, ipilimumab, and pembrolizumab have become integral components in the therapeutic arsenals for over 20 different cancer types [1,2,3]. As of May 2024, this evolution is underscored by the approval of 13 ICIs by the U.S. Food and Drug Administration (FDA), including the groundbreaking LAG-3 antibody relatlimab-rmbw, which, in conjunction with the PD-1 inhibitor nivolumab, is now used in the treatment of unresectable or metastatic melanoma in patients aged 12 years and older [4].

While ICIs have demonstrated remarkable clinical efficacy, they also bring about a distinctive spectrum of severe and occasionally life-threatening adverse events that demand the attention of both emergency physicians and primary oncologists [5,6,7,8]. These adverse events can affect nearly every organ and include cardiovascular [9,10,11,12], neurological, and neuromuscular adverse events [13,14,15,16]. Among the cardiovascular immune-related adverse events (irAEs), ICI-associated myocarditis is of particular concern, given its association with an alarming nearly 50% mortality rate and serious cardiovascular complications occurring in up to 46% of affected patients [17,18,19]. Further, to prevent continued toxicity, interrupting ICI treatment is necessary [20,21], which influences the progression of the cancer itself and alters the patient’s outcome. The impact of ICI-associated myositis is equally noteworthy, with one study revealing a 21% fatality rate and almost half of the affected patients experiencing prolonged hospitalization or severe complications [22]. Approximately 1% of ICI-treated patients develop myocarditis, with 25% of these individuals simultaneously developing myositis and 11% experiencing concurrent myasthenia gravis [17,18]. The occurrence of all three conditions in combination is exceptionally uncommon, with its documentation primarily confined to case reports and case series within the existing literature [17,23,24,25,26,27,28,29,30,31,32,33].

This emerging and potentially lethal toxicity syndrome characterized by the overlapping conditions of ICI-associated myocarditis, myositis, and myasthenia gravis (MMM) may not be widely recognized among emergency clinicians and other acute care practitioners [29]. This comprehensive review aims to raise awareness of this potentially life-threatening triad of irAEs and to provide insights into clinical management considerations required when patients present with this complex syndrome in emergency department or urgent care settings. By enhancing understanding and recognition of MMM, this review seeks to improve outcomes through timely and appropriate intervention strategies.

## 2. Materials and Methods

This systematic review was registered in the Open Science Framework (OSF) with the identifier (DOI: 10.17605/OSF.IO/M7YDS). We present the results of our search according to the Preferred Reporting Items for Systematic Reviews and Meta-Analysis (PRISMA) guidelines [34]. A systematic search of PubMed and Embase was conducted for all English language publications up to 1 August 2023 that reported ICI-associated concurrent myocarditis, myositis, and myasthenia gravis. The search strategy included terms such as myositis, myocarditis, myasthenia gravis, immune checkpoint inhibitor, PD-1, PDL-1, and CTLA-4. The full search strategy, including the terms and medical subject headings used, is shown in Supplemental Table S1. Additional reports were identified by reviewing the references of the final included articles.

Following the elimination of duplicate records in the initial identification phase, two independent reviewers, DNL and PPK, conducted a blind and independent assessment and screening of the reports. This assessment involved a review of the records’ titles and abstracts and was facilitated by using the Rayyan platform for systematic reviews [35]. Exclusion criteria were as follows: nonoriginal articles, including reviews, editorials, opinion pieces, and commentaries; studies that did not evaluate humans; studies that involved medications other than ICIs; and studies that did not report the triad of myocarditis, myositis, and myasthenia gravis as concurrent adverse events. Disagreements between the two reviewers were resolved by consensus. Following this, the full text of the selected reports from the screening phase was retrieved and blindly evaluated by two independent reviewers (DNL and AQ) to assess for eligibility, excluding reports with (1) no concurrent MMM cases, (2) reports with no individual or insufficient data on the MMM cases, (3) nonoriginal articles, (4) abstract or poster only, and (5) cases identified as duplicates by the reviewers. A comprehensive quality appraisal of the final identified studies was then conducted by NEM and AQ utilizing the Joanna Briggs Institute (JBI) quality appraisal tools [36,37]. Disagreements were resolved by consensus. Data extraction was then performed on the included studies by three authors (PPK, TDN, and PC). The following information was collected: patient age, sex, cancer type, ICI used, presenting signs and symptoms, time to presentation of concurrent adverse events in days, patient disposition and outcomes, treatment of irAEs, and diagnostic approach, including clinical, laboratory tests, and imaging.

Descriptive statistics and qualitative synthesis were used to summarize and report the main results. Continuous variables were reported as median and ranges, while categorical variables were reported as counts and percentages. All analyses were performed using R software version 4.3.3 (The R Foundation, http://www.r-project.org [accessed on 29 May 2024]).

## 3. Results

**Figure 1:**
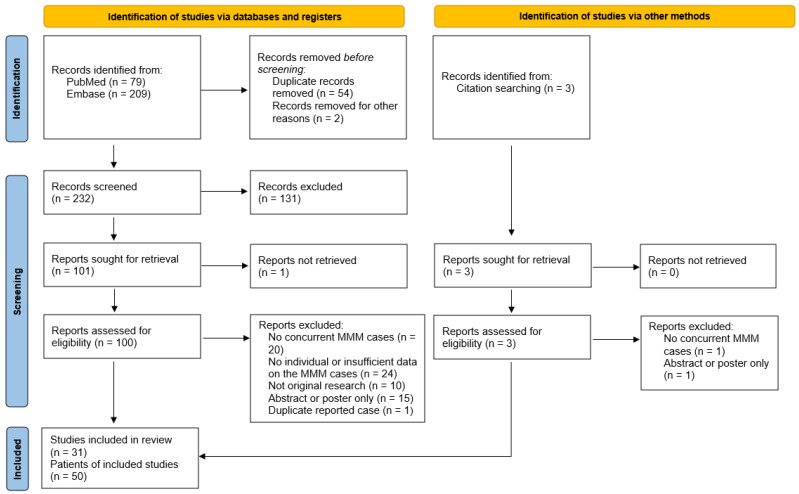
PRISMA flow diagram highlighting the inclusion and exclusion of studies at each step and the final number of studies included. MMM, myocarditis, myositis, and myasthenia gravis.

The initial electronic search yielded 288 references. An additional three references were identified via a citation search. Out of 232 references screened from the initial electronic search, 100 were assessed for eligibility, with the additional 3 references found via citation search. The PRISMA diagram demonstrating the steps for the literature review and the selection criteria is shown in Figure 1 [34].

A total of 31 eligible studies were included in our analysis [17,23,24,25,26,29,30,32,33,38,39,40,41,42,43,44,45,46,47,48,49,50,51,52,53,54,55,56,57,58,59]. Of the included studies, 25 were appraised as case reports, and 6 were appraised as case series [23,29,44,49,51,54]. Supplemental Table S2 summarizes the main characteristics and outcomes of the patients in the final included studies. The studies included 50 patients who developed the triad of myocarditis, myositis, and myasthenia gravis after receiving ICI therapy. The patients’ clinical and demographic characteristics are summarized in Table 1. Of the 50 patients with cancer, the majority were diagnosed with melanoma (28.0%), lung cancer (20.0%), renal cancer (14.0%), and thymoma (12.0%). The male-to-female ratio was about 2:1, and the median age was 70 years (interquartile range: 65–75 years). PD-1 inhibitors, including pembrolizumab (40.0%) and nivolumab (30.0%) were the most common ICIs used. CTLA-4 inhibitor ipilimumab was also frequently used (16.0%). In-hospital mortality was reported in 19 patients (38.0%).

Table 2 presents a comprehensive list of the presenting signs and symptoms for all patients analyzed. More than half (58.0%) of the patients had ptosis upon presentation. Other frequent presenting signs/symptoms were dyspnea (48.0%), diplopia (42.0%), myalgia (36.0%), muscle weakness (28.0%), or dysphagia (26.0%).

The time from ICI initiation to the occurrence of the MMM syndrome is reported in Supplemental Table S2. The median time was 21 days (interquartile range: 15–28), with the time occurrence ranging from 3 to 132 days.

All patients were suspected to have ICI-associated myocarditis based on clinical presentation and laboratory biomarkers, and confirmation using cardiac magnetic resonance or endomyocardial biopsy was obtained in 7 and 8 patients, respectively (1 patient had both). Forty-two patients were diagnosed with myositis based on laboratory findings (creatinine kinase). Myasthenia gravis was commonly diagnosed through clinical presentation, with 17 patients having abnormal levels of related antibodies. As summarized in Table 3, almost all patients (98%) were treated with corticosteroids for the irAEs. Other treatments included intravenous immunoglobulin (52.0%), plasma exchange (36.0%), pyridostigmine (20.0%), and monoclonal antibodies (rituximab and infliximab). The majority of the patients had more than one line of treatment at the same time (either concurrently or sequentially). Treatment strategies and clinical outcomes for each case are summarized in Table 4.

## 4. Discussion

In patients with cancer treated with ICI who present to the emergency department with suspected adverse events associated with their therapy, early recognition and prompt diagnostic strategies targeting irAEs are crucial, as some patients can quickly deteriorate leading to significant morbidity and mortality. Although some irAEs are uncommon, happening in less than 1% of cancer patients treated with ICI, these events could be fatal and require a proactive approach.

The results of this systematic review underscore the clinical significance of the ICI-induced MMM overlap syndrome, especially considering the concerning in-hospital mortality rate of 38.0%. Recent publications further reinforce our findings, emphasizing the ongoing emergence of cases and highlighting the importance of early recognition and prompt intervention in managing these complex irAEs [60,61,62,63,64,65,66,67,68,69,70,71,72]. These findings highlight the urgency of early recognition and effective management of these irAEs, particularly in the emergency department or urgent care settings, where timely interventions can be lifesaving [71]. The array of reported symptoms, which include dyspnea, ptosis, diplopia, myalgia, muscle weakness, and dysphagia, provides essential clinical insights that are crucial for enabling emergency physicians and other acute care clinicians to recognize potential cases with greater efficiency. Early identification and management of irAEs have been reported to lead to decreased morbidity and mortality [63,73]; thus, understanding and identifying the diverse presentation of this overlap syndrome is crucial for timely diagnosis and appropriate intervention. This is particularly important considering that studies have shown that both residents and attending physicians across various specialties often feel uncomfortable with the management and treatment of irAEs due to ICIs [74,75,76]. By raising awareness and enhancing the ability of healthcare professionals to promptly identify and manage this complex syndrome, we hope patient outcomes can be significantly improved, thereby reducing the risk of severe complications and mortality associated with this condition.

Our review further reveals that the diagnosis of ICI-associated MMM primarily relies on clinical presentation and laboratory biomarkers. In the case of myocarditis, cardiac magnetic resonance is considered the preferred imaging modality, and endomyocardial biopsy is the gold standard for diagnosis [77]. Although most of the reviewed cases had one of these two diagnostic tests performed to confirm the diagnosis, performing these tests presents a challenge in the emergency department, where rapid decision making is critical. Furthermore, even outside the emergency department or urgent care settings, there are several limitations that can prevent a patient from undergoing an invasive endomyocardial biopsy, such as patient clinical stability or the desire for such an invasive procedure. Similarly, although cardiac magnetic resonance imaging is not as invasive and is highly effective, factors such as contraindication to contrast agents and the presence of metallic implants or objects within patients’ bodies may pose significant challenges to its successful implementation. Contraindications to contrast agents, including allergies or compromised renal function, can limit their safe administration, thereby hindering the diagnostic utility of cardiac.

Cardiovascular adverse events after ICI including myocarditis can present to the emergency department or urgent care center for care [78]. Understanding the diagnostic approach to these events will enable better care for this cancer patient population [78,79]. Recently, the International Cardio-Oncology Society published a consensus statement in 2021 that allows for a clinical diagnosis of myocarditis [80]. The clinical diagnosis includes a new or significant troponin elevation from baseline with cardiac magnetic resonance results diagnostic for acute myocarditis or, alternatively, a troponin elevation with two of the following: clinical syndrome, ventricular arrhythmia or a new conduction system disease, decline in cardiac function, other irAEs (in particular, myositis, myasthenia gravis, or myopathy), and exclusion of acute coronary syndrome or acute infectious myocarditis [80]. In addition, a recent retrospective, multicenter study found that elevated kinase isoenzyme-MB (CK-MB), cardiac troponin-I levels, and neutrophil-to-lymphocyte ratio were all independent risk factors for the development of ICI-associated myocarditis [81]. Additionally, a biomarker-based algorithm for the diagnosis of ICI-related myocarditis has been proposed based on the role of biomarkers such as cardiac troponin and creatine phosphokinase [81,82]. This algorithm divides patients undergoing ICI therapy who present with symptoms suggestive of ICI-related myocarditis and an abnormal troponin into those that present more than 60 days from the first ICI infusion and those less than 60 days. When the first ICI infusion occurred more than 60 days prior and the patient has normal creatine phosphokinase (CPK) levels with rapidly declining troponin levels, the likelihood of ICI-related myocarditis is very low, and other causes of myocardial injury should be considered. Conversely, if ICI therapy began within the past 60 days and the patient exhibits abnormal CPK levels alongside stably elevated or rising troponin levels, the probability of ICI-related myocarditis is high. In such cases, after excluding acute coronary syndrome, immunosuppression should be strongly considered [82]. Despite the push for biomarker-based algorithms, clinicians must be aware of the potential limitations of laboratory testing, such as troponin T being falsely elevated in patients with myositis, and should consider early consultation with specialists for invasive procedures in complex or equivocal cases.

When it comes to myositis, there are currently no established, evidence-based diagnostic criteria, but workup and evaluation should include creatine kinase, aldolase, lactate dehydrogenase, alanine, and aspartate transaminase levels, as well as inflammatory biomarkers such as erythrocyte sedimentation rate (ESR) and C-reactive protein [83]. Other diagnostic methods, such as electromyography, magnetic resonance imaging, or muscle biopsy, can be considered but are not readily available in the emergency department [21]. The lack of these more advanced diagnostics should not prevent the emergency clinician from treating a suspected ICI-related myosis. The typical presentation of ICI-related myositis can include subacute muscle weakness with increased CK levels; however, normal or only slightly elevated CK levels do not rule out ICI-related myositis [84]. It is also important to consider that during its peak, ICI-related myositis can present with bulbar symptoms similar to those present in myasthenia gravis and respiratory failure may even occur from only ICI-related myosis [85]. Our review showed that over 50% of patients with this triad presented with ptosis, nearly half with dyspnea and over 40% exhibited diplopia. Thus, it is important to keep myasthenia gravis and myositis both in the differential diagnosis of muscle weakness, bulbar symptoms, and even dyspnea in patients presenting with such symptoms [86].

Similar to myositis, the diagnosis of ICI-related myasthenia gravis necessitates a multifaceted approach incorporating laboratory and clinical assessments. Although laboratory testing for antiacetylcholine receptors and antistriated muscle antibodies can aid diagnosis, their accessibility in emergency departments is limited. Furthermore, the absence of these antibodies does not definitively exclude the syndrome, given that only around two-thirds of patients with ICI-related myasthenia gravis test positive for antiacetylcholine receptor antibodies [21,86]. A recent study utilizing Vigibase, the World Health Organization’s pharmacovigilance database, highlights the contrasting neurologic adverse event (AE) profiles between patients receiving ICIs and the broader database [87]. Among the extensive dataset, ICIs were associated with a markedly higher percentage of myasthenia gravis reports compared with the full database. Specifically, myasthenia gravis was reported in 0.47% of ICI cases, in contrast to only 0.04% in the full database. Notably, myasthenia gravis associated with ICIs exhibited distinct characteristics, including higher fatality rates, an earlier onset, and increased frequency of concurrent myocarditis and myositis. In contrast, other neurologic AEs showed lower fatality rates (6–12% vs. ~20%) and a later onset with a median of 61–80 days, with minimal overlap in symptoms [87]. Finally, rapid progression to respiratory failure has been documented in up to 50% of cases of ICI-related myasthenia gravis, underscoring the criticality of prompt identification and management to avert deterioration [31,87]. These findings highlight the imperative nature of discerning the unique clinical manifestations and trajectories associated with myasthenia gravis induced by ICIs, particularly in the context of their potential for rapid and severe respiratory compromise. Such insights are paramount for optimizing patient outcomes and informing clinical management strategies in this burgeoning domain of immune-related adverse events [84].

Corticosteroids emerged as the cornerstone of treatment for irAEs in almost all of the cases studied, aligning with clinical practice guidelines [21]. It is advisable to commence treatment promptly in the emergency department, even prior to the completion of confirmatory diagnostic testing [88]. However, our review also highlights the use of additional treatment modalities such as intravenous immunoglobulin, plasma exchange, pyridostigmine, and monoclonal antibodies. The selection of a secondary immunosuppressive agent is frequently guided by past experiences with autoimmune conditions that share similarities with the specific irAE in question, despite limited empirical data driving these choices. While adequate immunosuppression is needed in treating irAEs, the association between immunosuppression and ICI efficacy continues to be further explored [89]. These therapeutic options further reflect the complexity of managing the MMM triad and underscore the importance of a multidisciplinary approach. Close collaboration between emergency physicians and oncologists, among other specialists, is essential to tailor treatment strategies to individual patient needs.

Additionally, the findings suggest a predominant association between the MMM triad and an underlying diagnosis of melanoma, as well as the use of specific ICIs, including pembrolizumab, nivolumab, and ipilimumab. Our findings are similar to those in a broader 2021 review by Pathak et al. encompassing patients with the MMM triad, as well as other overlapping adverse events [90]. While the reasons for these associations remain unclear, further research is warranted to elucidate whether they are related to the biology of melanoma or specific immunological mechanisms triggered by these ICIs. This information could guide risk assessment and surveillance strategies in patient populations with these characteristics.

The rarity of the MMM triad, coupled with the expanding use of ICIs in various cancer types, raises important questions about its potential future prevalence. Further research is needed to investigate the underlying pathophysiological mechanisms and the potential influence of factors such as genetic predisposition and immune responses. This knowledge could inform risk stratification and the development of early diagnostic and therapeutic interventions, ultimately improving patient outcomes and reducing associated morbidity and mortality.

### Limitations

Several limitations need to be considered in the interpretation of our systematic review. First, our analysis primarily relied on case reports and case series, which can introduce publication bias and limit generalizability. The inherent heterogeneity in the quality and depth of data from these sources posed challenges in conducting a meta-analysis and drawing consistent conclusions. Moreover, the potential underreporting may hinder the establishment of a causal relationship between ICI treatment and the MMM triad, affecting our ability to make strong recommendations regarding management. The lack of comprehensive data on treatment efficacy and outcomes further limits the depth of our findings. Additionally, a focus on English-language publications and potential regional bias may have restricted the inclusivity of a global perspective. Finally, our review is based on the literature available up to a specific point in time, and the rapidly evolving landscape of ICI therapy and clinical understanding may have introduced temporal limitations. Notwithstanding the outlined limitations, this systematic review serves as a comprehensive compilation of the available evidence on a syndrome primarily reported in case reports and series, enhancing clinical recognition, and offering a thematic synthesis of its clinical manifestations, diagnostics, and therapeutics. The review underscores knowledge gaps and emphasizes the need for further research, which can guide future investigations and clinical guidelines, ultimately leading to improved patient care.

## 5. Conclusions

Awareness of the ICI-induced MMM overlap syndrome is paramount, given its high in-hospital mortality rate. Timely recognition of a diverse array of symptoms is crucial, guiding healthcare providers, especially emergency physicians, in their diagnostic and management efforts. The complex nature of MMM necessitates a multidisciplinary approach to patient care. Collaboration between specialists across various disciplines, including oncology, cardiology, rheumatology, and emergency medicine, is essential for navigating the diagnostic and therapeutic challenges posed by this syndrome. By pooling expertise and resources from different fields, healthcare teams can formulate individualized management strategies tailored to each patient’s unique clinical presentation and underlying risk factors. Raising awareness of the ICI-induced MMM overlap syndrome and fostering collaboration among healthcare providers are essential steps toward improving patient outcomes.

## Figures and Tables

**Table 1:** Clinical and demographical characteristics for patients with concurrent ICI-associated myocarditis, myositis, and myasthenia gravis triad.

Characteristic	*n* (%)
Total	50
Age, median (IQR), years	70 (65–75)
Sex	
Female	16 (32)
Male	34 (68)
Cancer type	
Melanoma	14 (28)
Lung cancer	10 (20)
Renal cancer	7 (14)
Thymoma	6 (12)
Bladder cancer	3 (6)
Colorectal cancer	2 (4)
Esophageal cancer	2 (4)
Cholangiocarcinoma	2 (4)
Head and neck cancer	1 (2)
Breast cancer	1 (2)
Prostate cancer	1 (2)
Sarcoma	1 (2)
Days from ICI to presentation, median (range)	21 (15–28)
Immune checkpoint inhibitor type ^1^	
Pembrolizumab	20 (40)
Nivolumab	15 (30)
Ipilimumab	8 (16)
Sintilimab	5 (10)
Durvalumab	4 (8)
Camrelizumab	1 (2)
Avelumab	1 (2)
Cemiplimab	1 (2)
Tremelimumab	1 (2)
Spartalizumab	1 (2)
Tislelizumab	1 (2)
Toripalimab	1 (2)
Reported in-hospital mortality	
No	31 (62)
Yes	19 (38)

^1^ Numbers do not add to 100% as some patients had more than one immune checkpoint inhibitor.

**Table 2:** Presenting signs and symptoms reported in patients with concurrent ICI-associated myocarditis, myositis, and myasthenia gravis, after immune checkpoint inhibitor therapy.

Sign/Symptom	*n* (%) ^1^
Ptosis	29 (58)
Dyspnea	24 (48)
Diplopia	21 (42)
Myalgia	18 (36)
Muscle weakness	14 (28)
Dysphagia	13 (26)
Fatigue	12 (24)
Dysphonia	6 (12)
Chest pain or tightness	4 (8)
Palpitations	4 (8)
Dizziness	4 (8)
Dysarthria	4 (8)
Extraocular muscle deficit	3 (6)
Neck weakness	3 (6)
Presyncope	3 (6)
Head droop	2 (4)
Blurred vision	2 (4)
Paresis	2 (4)
Rash	2 (4)
Gait problems	1 (2)
Facial droop	1 (2)
Arthralgia	1 (2)
Malaise	1 (2)
Fever	1 (2)
Other visual problems	6 (12)
Others	8 (16)

^1^ Numbers do not add to 100% as some patients had more than one sign or symptom at the same time.

**Table 3:** Treatment modalities for patients with concurrent ICI-associated myocarditis, myositis, and myasthenia gravis after immune checkpoint inhibitor therapy.

Treatment	*n* (%) ^1^
Corticosteroids	49 (98.0)
Intravenous immunoglobulin	26 (52.0)
Plasma exchange	18 (36.0)
Pyridostigmine	10 (20.0)
Mycophenolate mofetil	9 (18.0)
Rituximab	7 (14.0)
Cyclophosphamide	4 (8.0)
Infliximab	4 (8.0)
Anti-thymocyte globulin	3 (6.0)
Plasmapheresis	2 (4.0)
Physostigmine	1 (2.0)
Alemtuzumab	1 (2.0)

^1^ Numbers do not add to 100% as some patients had more than one medication at the same time.

**Table 4:** Treatment strategies and clinical response reported in patients with concurrent ICI-associated myocarditis, myositis, and myasthenia gravis after immune checkpoint inhibitor therapy.

Author	Patient Sequence	Age, Years	Treatment Strategy ^1^	Clinical Response ^1^
Shirai et al. [30]	1	83	3 days of methylprednisolone 1000 mg/day, then tapered prednisolone (1 mg/kg/day to 30 mg/day) and 4 cycles of plasma exchange	ECG and blood tests improved shortly after therapy initiation. Wide QRS and AV block improved in 3 days. CK and troponin-T levels decreased. Ptosis, ophthalmoplegia, and neck weakness improved after 6 weeks.
Esfahani et al. [41]	1	71	3 days of methylprednisolone 1 g/day, then 200 mg/day. Mycophenolate mofetil 1 g twice/day. Plasmapheresis daily for 5 days. Rituximab IV 375 mg/m² weekly. Alemtuzumab 30 mg on day 18. Weaned off rituximab, glucocorticoids, and mycophenolate mofetil over 4 weeks.	Initial response with improved biochemical variables by day 7. Developed cardiac arrhythmias on day 18. Resolution of myocarditis and myositis by day 28. Weaned off all treatments by day 50.
Fazel et al. [17]	1	78	Methylprednisolone IV: 75 mg (day 1), 125 mg (days 2–3), 1000 mg (days 4–6), 150 mg (day 7), 75 mg (day 8). IVIG 2 mg/kg (days 5–6). Plasmapheresis 1 cycle (day 7).	Biomarkers decreased. Muscle weakness slightly improved. Bulbar symptoms worsened, leading to discharge to hospice.
Konstantina et al. [45]	1	30	Prednisolone 2 mg/kg, pyridostigmine, IVIG (400 mg/kg for 5 days), rituximab 375/m² weekly	Developed eyelid drop, diplopia, respiratory failure, liver transaminases increase. Intubated, ICU 30 days. Improved with rituximab, weaned from ventilation. Developed septic shock, died on day 64.
Todo et al. [32]	1	63	Prednisolone 1 mg/kg (60 mg/day), tapered over 321 days	Biomarkers gradually decreased, and symptoms improved.
Arora et al. [23]	1	70	IV steroids 1 mg/kg initially, then ATG and increase in steroids to 1 g methylprednisolone. MMF and cyclophosphamide on day 5, plasmapheresis on day 6	Progressive cardiac abnormalities, cardiac arrest, intubation for respiratory failure, died after unsuccessful resuscitation.
	2	79	IV steroids 1 mg/kg, increased to 1 g/day, ATG and MMF on day 1, cyclophosphamide on day 3, IVIG for MG	Cardiac biomarkers decreased, a permanent pacemaker was placed, and there was no improvement in generalized weakness or ophthalmoplegia. Complications including GI bleed and PE, transitioned to hospice.
	3	61	IV steroids 2 mg/kg, pyridostigmine, MMF added for progressive troponin increase	Troponin decreased with MMF. After discharge, she returned to the hospital with chest pressure, developed SIADH hypercapnic respiratory failure, and transitioned to comfort measures.
	4	67	IV steroids 2 mg/kg, MMF, increased to 1 g methylprednisolone, ATG, plasmapheresis	Developed hypercapnic respiratory failure, troponin rose while CK and transaminases decreased, transitioned to comfort measures.
	5	70	IV steroids, plasmapheresis, and initial steroids were increased to methylprednisolone 1 g per day. Infliximab	The patient required intubation for respiratory failure. Developed upper GI bleeding from immune-related gastritis, and Infliximab was given. Deteriorated with dysphagia, dyspnea, required intubation, GI bleeding, persistent respiratory failure, transitioned to comfort measures.
	6	89	IV steroids 1 mg/kg.	Multiple episodes of nonsustained ventricular tachycardia and high-degree AV block transitioned to comfort measures.
Fazal et al. [25]	1	82	Started on IV immunoglobulin 0.4 g/kg/day for 5 days. Upon rapid deterioration, high-dose IV methylprednisolone 1 g was started. Dual antiplatelets were given for troponin rise. He was then transitioned to oral prednisolone, and pyridostigmine was initiated.	Showed initial improvement and was extubated within 48 h. Ptosis and dysarthria continued. Reintubated due to respiratory failure. Experienced GI bleeding, fevers, hemodynamic deterioration, and death.
Jejakumar et al. [26]	1	86	IV methylprednisolone 1 g, plasma exchange for 5 days, continued high-dose methylprednisolone, IV immunoglobulin	Intubated on arrival, rising troponin levels, worsening kidney function, required renal replacement therapy. Died from hyperkalemia and severe metabolic acidosis despite resuscitation efforts.
Luecke et al. [47]	1	67	High-dose systemic glucocorticoids, 5 cycles of plasmapheresis, pyridostigmine	Showed no clinical improvement despite immunosuppressive treatment. Required intubation and mechanical ventilation, died 18 days after ICU admission.
Xing et al. [33]	1	66	Methylprednisolone (MP) 2 mg/kg/day and IV immunoglobulin 400 mg/kg/day for 5 days, temporary pacemaker for 2 days. Adjusted to MP 500 mg/day for 5 days, then tapered, and pyridostigmine bromide 120 mg twice daily.	Intubated after NIPPV. Peripheral limb and eye-opening symptoms improved, serum CPK normalized, anti-AChR-Ab decreased. Respiratory muscle weakness persisted. After two PLEX courses, anti-AChR-Ab normalized, breathing improved. Now on pyridostigmine, mechanical ventilation 12 h/day, and rehab
Bawek et al. [40]	1	68	Pyridostigmine 60 mg TID, IVIG. Due to continued deterioration, on day 8, 1000 mg methylprednisolone (mPSL) IV daily for 3 days was started, followed by prednisone taper	Initial symptom improvement with mPSL, but developed intractable diarrhea (IVIG-related), increased oxygen requirement, multiple organ failure, and possible heparin-induced thrombocytopenia. Transferred to hospice care.
Cham et al. [24]	1	72	On admission corticosteroid use was considered, but the patient declined because of existing central serous retinopathy. Due to his declining respiratory status, he was transferred to the ICU and intubated on day 9. At that point, high-dose corticosteroids at 1 mg/kg/day and plasmapheresis were started on hospital day 10, completing 5 rounds.	Developed progressive axial weakness and respiratory decline, intubated on day 9, tracheostomy and PEG placement required. Transferred to long-term care facility on day 36 due to ventilation dependence.
Lipe et al. [29]	1	49	Steroids, PLEX, IVIG	Alive at discharge.
	2	67	Steroids, IVIG, Cellcept	Alive at discharge.
	3	70	Steroids, infliximab, PLEX	Death.
	4	81	Steroids, infliximab, rituximab, PLEX	Alive at discharge.
	5	75	Steroids, PLEX	Alive at discharge.
	6	66	Steroids, Infliximab, Rituximab, PLEX	Alive at discharge.
	7	74	Steroids, infliximab, PLEX, IVIG	Death.
Luo et al. [48]	1	47	IV immunoglobulin (0.4 g/kg/day for 5 days), followed by pulse methylprednisolone (500 mg, then 250 mg/day for 5 days), then oral prednisolone (60 mg/day for 4 weeks, tapering to 50 mg/day)	Developed type II respiratory failure, intubated, and mechanically ventilated. Third-degree AV block treated with a pacemaker. Improved limb strength after 69 days, but still had difficulty weaning from the ventilator. Transferred for rehab; off mechanical ventilation and on noninvasive ventilation after 1 month.
Yang, Xu et al. [57]	1	66	High-dose IV steroids (500 mg/day for 3 days, 250 mg/day for 3 days, 120 mg/day for 3 days, then tapered), IV immunoglobulin (25 g/day for 5 days). Additional treatments included coenzyme Q10, trimetazidine, recombinant human brain natriuretic peptide, diuretics, cetirizine, calamine lotion, magnesium isoglycyrrhizinate, nadroparin calcium, insulin, cefoperazone sulbactam, and albumin infusion	Symptoms resolved, and examination gradually normalized.
Yang, Chen et al. [58]	1	33	Methylprednisolone (2 mg/kg/day), human immunoglobulin (20 g/day for 5 days), and pyridostigmine (180 mg/day). Oral prednisone tapered over 6 months.	Symptoms significantly improved within days; LV normalized and QRS complexes returned to normal.
Bai et al. [39]	1	69	Methylprednisolone sodium succinate (120 mg/day for 5 days, reduced to 80 mg/day) on the 6th day. Due to deterioration, the steroids were restarted at 120 mg with tapering. Oral pyridostigmine bromide (30 mg qid, tapered). Immunoglobulin injections for 1 week.	Initially developed lower extremity weakness and respiratory failure. Transferred to the ICU and intubated. Diagnosed with ventilator-associated pneumonia. Weaned off the ventilator after 2 weeks. Gradual reduction in glucocorticoid dosage, improved myocardial biomarkers, and muscle strength. Treated with oral prednisone (15 mg daily, tapered) and pyridostigmine bromide (30 mg three times a day) during recovery.
Hyun et al. [44]	1	55	IV methylprednisolone 1 g/day for 3 days	death
	2	64	IV methylprednisolone1 g/d for 5 d, plus IVIG at 2 g/kg	Discharged alive without significant disability,
Nakagomi et al. [51]	1	77	Plasma exchange, IVIG (400 mg/kg/day for 5 days) and increasing oral prednisone. Steroid pulse therapy with IV methylprednisolone (1 g/day for 3 days), followed by a second and third pulse due to continuous increasing troponin levels.	MG and myositis symptoms improved with declining CK levels after initial therapy. However, myocarditis worsened. Troponin levels, although initially decreased, kept re-elevating, thus needing several pulses of MP. The patient was discharged alive.
	2	73	Pulse therapy with IV methylprednisolone (1 g/day for 3 days), followed by oral prednisone. Two additional steroid pulses were required due to persistent troponin elevation.	Rapid recovery from myositis and MG with reduced CK and improved eyelid ptosis, but increased myocarditis activity (elevated troponin and persistent chest discomfort). Discharged on day 36.
Saishu et al. [52]	1	55	Initially treated with IV immunoglobulin and prednisolone (20 mg/day) before definitive diagnosis and based on presenting symptoms only. Continued with immunoglobulin, corticosteroids (methylprednisolone and prednisolone), and plasma exchange (five times).	Improved muscle weakness, ptosis, ocular motility disorder, and CK levels. Despite initial ICU admission and intubation due to respiratory failure, symptoms gradually improved, and the patient could walk with a cane after rehabilitation.
Soman et al. [53]	1	73	Prednisolone, immunoglobulin infusion, physostigmine.	Developed complete heart block; required isoproterenol. Pacemaker implantation failed. Desaturated, needed advanced noninvasive ventilation. Raised right hemidiaphragm due to phrenic nerve palsy. Died on day 7 of admission.
Wai Siu et al. [54]	1	73	Prednisone Methylprednisolone PLEX IVIG Mycophenolate	Resolution of toxicity.
	2	74	Prednisone Methylprednisolone Mycophenolate	Resolution of toxicity.
	3	73	Methylprednisolone Prednisone IVIG	Resolution of toxicity.
Wang et al. [55]	1	65	Methylprednisolone (1 g/day for 3 days, then taper) and IVIG (0.4 g/kg/day for 3 days).	Myocardial enzymes decreased gradually; biomarkers normalized over 20 days; discharged with intermittent ventilator support.
Wu et al. [56]	1	48	Pyridostigmine, IV methylprednisolone (1 mg/kg/day, tapered every 3 days), normal saline hydration for 5 days. Continued with Pyridostigmine 60 mg 1 tablet/day and dexamethasone 4 mg 2 tablet per day after discharge	Symptoms gradually improved; a 1-month follow-up showed normal eye movement and reduced diplopia.
Yin et al. [59]	1	71	Methylprednisolone (500 mg/day for 5 days, then taper) and IVIG (0.4 g/kg/day for 5 days). Tacrolimus (3 mg/day) added due to weakness and soreness bilateral extremities.	Significant clinical improvement: biomarker levels declined; discharged.
Ahdi et al. [38]	1	58	IVIG (667 mg/kg/day for 3 days, total 2 g/kg) and pyridostigmine.	Symptoms and liver function improved; discharged on oral prednisone.
Giovannini et al. [42]	1	65	Intravenous methylprednisolone (60 mg/day) and oral pyridostigmine (60 mg, three times daily). Anticoagulant therapy initiated.	On day 2, developed dyspnea, atrial fibrillation, and severe hypoxemia. Despite noninvasive ventilation, dialysis, and resuscitation, the patient died from ventricular tachycardia and fibrillation.
Golec et al. [43]	1	74	Methylprednisolone (1 mg/kg, escalated to 1000 mg/day), plasma exchange (PLEX), mycophenolate mofetil. PLEX was initially held but later resumed.	Developed tamponade (treated with pericardiocentesis), complete heart block (treated with pacemaker), worsening myasthenia, intubation, and 6 additional PLEX sessions. He transitioned to comfort care and died.
Lin X. et al. [46]	1	51	Methylprednisolone (500 mg/day, reduced to 250 mg/day), IVIG (5 g/day), pyridostigmine, and low-flow oxygen.	Symptoms of overlap syndrome improved; respiratory weakness and biomarkers (cTnI and CK) normalized. Discharged on day 18.
Marco et al. [49]	1	77	Corticosteroids, PLEX, mechanical ventilation	Death at 60 days follow-up.
	2	78	Corticosteroids, immunoglobulins, plasma exchange, Rituximab, mechanical ventilation	Death at 133 days follow-up.
	3	70	Corticosteroids, immunoglobulins, Ciclofosfamide, mechanical ventilation	Death at 53 days follow up.
	4	85	Corticosteroids, immunoglobulins	Death at 30 days follow up.
Masood et al. [50]	1	75	On day 1, the patient received 500 mg IV methylprednisolone, followed by 1 g IV methylprednisolone (3 doses), IVIG (2 g/kg over 5 days), and Cyclophosphamide (500 mg IV) on day 10. On day 26, 1 g rituximab (with a repeat dose in 2 weeks) was given. The patient continued with IVIG (0.4 g/kg/day for 5 days) and monthly IVIG for 3 months.	Developed RBBB, complete heart block, asystole, and cardiac arrest; required a permanent pacemaker. Progressive muscle weakness, dysphagia, and respiratory failure. Tracheal decannulation on day 133. Post-discharge remained on prednisolone with normal muscle strength but progressed melanoma with cutaneous metastases.
	2	77	Three pulses of 1 g IV methylprednisolone, five days of IVIg (2 g/kg). On day 6 the patient received 500 mg IV cyclophosphamide (plus 5 cycles every 2 weeks), IV rituximab (days 7 and 21), and monthly IVIG for 5 months.	Intubated and received a pacemaker and tracheostomy on day 9. CK improved; tracheostomy decannulated on day 119. Discharged home and maintained on Prednisolone (5 mg daily).

^1^ Specific dosing, medication, and clinical outcome details may not be specified if they were not provided in the referenced manuscript. anti-AChR-Ab: antiacetylcholine receptor antibody; ATG: Antithymocyte globulin; AV: atrioventricular block; CK: creatinine kinase; g: gram; GI: gastrointestinal; ICU: intensive care unit; IV: intravenous; IVIG: intravenous immunoglobulin; kg: kilogram; LV: left ventricle; m^2^: meter squared; mg: milligram; MG: myasthenia gravis; MMF: Mycophenolate mofetil; MP: methylpredsinolone; mPSL: methylprednisolone pulse therapy; PEG: percutaneous endoscopic gastrostomy; PLEX: Plasma exchange; RBBB: right bundle branch block; SIADH: syndrome of inappropriate antidiuretic hormone.

## Data Availability

All data used are drawn from the literature and available in PubMed and/or Embase.

## Supplementary Materials

The following supporting information can be downloaded at https://www.mdpi.com/article/10.3390/diagnostics14161794/s1, Table S1: Search strategy and search terms used to identify the studies via databases between 1 January 2010, and 1 August 2023; Table S2: Summary of the main characteristics and outcomes of the patients in the final included studies.
