# Supplementary material for: Myocarditis, Myositis, and Myasthenia Gravis Overlap Syndrome Associated with Immune Checkpoint Inhibitors: A Systematic Review
Source: Diagnostics (Basel). 2024 Aug 16;14(16):1794. doi: 10.3390/diagnostics14161794 (PMC11353298; doi:10.3390/diagnostics14161794)
Supplement: Supplementary file 1 [file diagnostics-14-01794-s001.zip › diagnostics-2942611-supplementary.pdf]

diagnostics

Supplementary Material

Tables

Table S1. Search strategy and search terms used to identify the studies via databases between January 1st, 2010 and August 1st 2023.

Database/Terms	Search Terms
Pubmed	(((myositis) AND (myasthenia)) AND (myocarditis)) AND ((((((((((((((((((((Immune Checkpoint Inhibitors[MeSH Terms]) OR (Immune checkpoint)) OR (immunotherapy)) OR (pd1)) OR (pd-1)) OR (pdl1)) OR (pd-l1)) OR (ctla4)) OR (ctla-4)) OR (checkpoint blocker)) OR (checkpoint inhibitor)) OR (atezolizumab)) OR (avelumab)) OR (cemiplimab)) OR (dostarlimab)) OR (durvalumab)) OR (ipilimumab)) OR (nivolumab)) OR (pembrolizumab)) OR (relatlimab)) OR (retifanlimab)) OR (tremelimumab))
Embase	Using AND or OR for the following: #1. ('immune'/exp OR immune) AND ('checkpoint'/exp OR checkpoint) AND ('inhibitor'/exp OR inhibitor); #2. 'myasthenia gravis'; #3. 'myocarditis'; #4. 'myositis'; #5. 'nivolumab' OR 'pembrolizumab' OR 'ipilimumab' OR 'atezolizumab' OR 'avelumab' OR 'cemiplimab' OR 'dostarlimab' OR 'durvalumab' OR 'relatlimab' OR 'retifanlimab' OR 'tremelimumab'
Terms	[MeSH]: Myocarditis, Myasthenia Gravis, Myositis, Immune Checkpoint Inhibitors<br>[Emtree]: myocarditis, myasthenia gravis, myositis, immune checkpoint inhibitor

Table S2. Summary of the main characteristics and outcomes of the patients in the final included studies.

Author	Year	Patient Sequence	Age, Years	Sex	Cancer Type	Immunotherapy Type	Days at Presentation or Symptom Onset	Reported Comorbidities	Reported in hospital outcome
Shirai et al. [30]	2018	1	83	Male	Melanoma	Pembrolizumab	25		Alive
Esfahani et al. [41]	2019	1	71	Female	Melanoma	pembrolizumab	18		Alive
Fazel et al. [17]	2019	1	78	Female	Melanoma	Nivolumab/ Ipilimumab	5	Hypertension, asthma, pulmonary embolism, and depression	Alive
Konstantina et al. [45]	2019	1	30	Female	Thymoma	Pembrolizumab	3		Death
Todo et al. [32]	2019	1	63	Male	Bladder	Pembrolizumab	34	Diabetes, colon cancer	Alive
Arora et al. [23]	2020	1	70	Male	Melanoma	Nivolumab/Ipilimumab	11	Hypertension, colon cancer	Death
		2	79	Male	Melanoma	Pembrolizumab	26	Chronic lymphocytic leukemia	Death
		3	61	Female	Breast	Durvalumab/ Tremelimumab	28		Death
		4	67	Female	Melanoma	Nivolumab/ Ipilimumab	14		Death
		5	70	Male	Renal	Nivolumab/ Ipilimumab	21	Hypertension, chronic kidney disease, atrial fibrillation	Death
		6	89	Male	Lung	Pembrolizumab	32	Hypertension, hyperlipidemia, coronary artery disease, chronic kidney disease, diabetes	Death
Fazal et al. [25]	2020	1	82	Male	Melanoma	Nivolumab	10	Diabetes, hypertension	Death
Jejakumar et al. [26]	2020	1	86	Male	Head and neck	Cemiplimab	21	Cutaneous carcinomas, hypertension, hyperlipidemia, coronary artery bypass graft, sick sinus syndrome	Death

								status, and chronic kidney disease	
Luecke et al. [47]	2020	1	67	Male	Lung	Pembrolizumab	14	Thymoma	Death
Xing et al. [33]	2020	1	66	Male	Lung	Sintilimab	25		Alive
Bawek et al. [40]	2021	1	68	Male	Melanoma	Nivolumab	21	Neuroendocrine carcinoma	Alive
Cham et al. [24]	2021	1	72	Male	Lung	Durvalumab	18	Prostate cancer, diabetes mellitus, hypertension, retinopathy, obstructive sleep apnea and chronic obstructive pulmonary disease	Alive
Lipe et al. [29]	2021	1	49	Female	Thymoma	Pembrolizumab	21		Alive
		2	67	Male	Lung	Durvalumab	28		Alive
		3	70	Male	Bladder	Pembrolizumab	25		Death
		4	81	Female	Renal	Nivolumab/ Ipilimumab	92		Alive
		5	75	Male	Sarcoma	Pembrolizumab	62		Alive
		6	66	Female	Renal	Nivolumab/ Ipilimumab	132		Alive
		7	74	Female	Melanoma	Nivolumab/ Ipilimumab	30		Death
Luo et al. [48]	2021	1	47	Female	Thymoma	Toripalimab	28		Alive
Yang, Xu et al. [57]	2021	1	66	Female	Colorectal	Sintilimab	15	Hypertension, diabetes	Alive
Yang, Chen et al. [58]	2021	1	33	Male	Thymoma	Sintilimab	34		Alive
Bai et al. [39]	2022	1	69	Male	Esophageal	Camrelizumab	23	Diabetes	Alive
Hyun et al. [44]	2022	1	55	Female	Thymoma	Pembrolizumab	15		Death
		2	64	Female	Thymoma	Pembrolizumab	12		Alive
Nakagomi et al. [51]	2022	1	77	Male	Renal	Pembrolizumab	21	Unstable angina	Alive
		2	73	Male	Renal	Nivolumab	15	Myocardial infarction	Alive
Saishu et al. [52]	2022	1	55	Female	Melanoma	Nivolumab	21	Thymoma	Alive
Soman et al. [53]	2022	1	73	Female	Lung	Pembrolizumab	21	Diabetes, Hypertension, chronic obstructive pulmonary disease, peripheral vascular disease	Death

Wai Siu et al. [54]	2022	1	73	Male	Renal	Nivolumab/ Ipilimumab	25	Hypertension, prostate cancer, osteoarthritis	Alive
		2	74	Male	Bladder	Avelumab	21	Hypertension, hyperlipidemia, diabetes, chronic kidney disease, coronary artery disease	Alive
		3	73	Male	Lung	Pembrolizumab	33	Hypertension, hyperlipidemia, atrial fibrillation, prostate cancer, and idiopathic peripheral neuropathy	Alive
Wang et al. [55]	2022	1	65	Male	Colorectal	Tislelizumab	20		Alive
Wu et al. [56]	2022	1	48	Male	Cholangiocarcinoma	Nivolumab	30		Alive
Yin et al. [59]	2022	1	71	Female	Cholangiocarcinoma	Sintilimab	7		Alive
Ahdi et al. [38]	2023	1	58	Male	Renal	Pembrolizumab	35	Non-alcoholic steatohepatitis	Alive
Giovannini et al. [42]	2023	1	65	Male	Melanoma	Pembrolizumab	12		Alive
Golec et al. [43]	2023	1	74	Male	Esophageal	Nivolumab	25		Death
Lin X. et al. [46]	2023	1	51	Male	Lung	Sintilimab	19	Hypertension	Alive
Marco et al. [49]	2023	1	77	Male	Lung	Spartalizumab	60		Death
		2	78	Male	Prostate	Pembrolizumab	19		Death
		3	70	Male	Melanoma	Nivolumab	15		Death
		4	85	Male	Lung	Durvalumab	37		Death
Masood et al. [50]	2023	1	75	Male	Melanoma	Pembrolizumab	14	Hypertension, hypercholesterolemia	Alive
		2	77	Male	Melanoma	Pembrolizumab	28	Hypertension, diabetes and psoriasis	Alive
